# Crystal structure of [1,1′:3′,1′′-ter­phenyl]-2′,3,3′′-tri­carb­oxy­lic acid

**DOI:** 10.1107/S2056989015015029

**Published:** 2015-08-22

**Authors:** Daniel A. Decato, Orion B. Berryman

**Affiliations:** a Department of Chemistry and Biochemistry, University of Montana, 32 Campus Dr., Missoula, Montana 59812, USA

**Keywords:** crystal structure, hydrogen bonding, meta-terphen­yl

## Abstract

The asymmetric unit of the title compound, C_21_H_14_O_6_, com­prises two symmetrically independent mol­ecules that form a locally centrosymmetric hydrogen-bonded dimer, with the planes of the corresponding carb­oxy­lic acid groups rotated by 15.8 (1) and 17.5 (1)° relative to those of the adjacent benzene rings. The crystal as a whole, however, exhibits a noncentrosymmetric packing, described by the polar space group *Pca*2_1_. The dimers form layers along the *ab* plane, being inter­connected by hydrogen bonds involving the remaining carb­oxy­lic acid groups. The plane of the central carb­oxy­lic acid group forms dihedral angles of 62.5 (1) and 63.0 (1)° with those of the adjacent benzene rings and functions as a hydrogen-bond donor and acceptor. As a donor, it inter­connects adjacent layers, while as an acceptor it stabilizes the packing within the layers. The ‘distal’ carb­oxy­lic acid groups are nearly coplanar with the planes of the adjacent benzene rings, forming dihedral angles of 1.8 (1) and 7.1 (1)°. These groups also form intra- and inter-layer hydrogen bonds, but with ‘reversed’ functionality, as compared with the central carb­oxy­lic acid groups.

## Related literature   

For a detailed discussion on local centers of symmetry in the space group *Pca*2_1_, see: Marsh *et al.* (1998 ▸). For the synthesis of the starting material 3,3′′-dimethyl-[1,1′:3′,1′′-terphen­yl]-2′-carb­oxy­lic acid, see: Du *et al.* (1986 ▸).
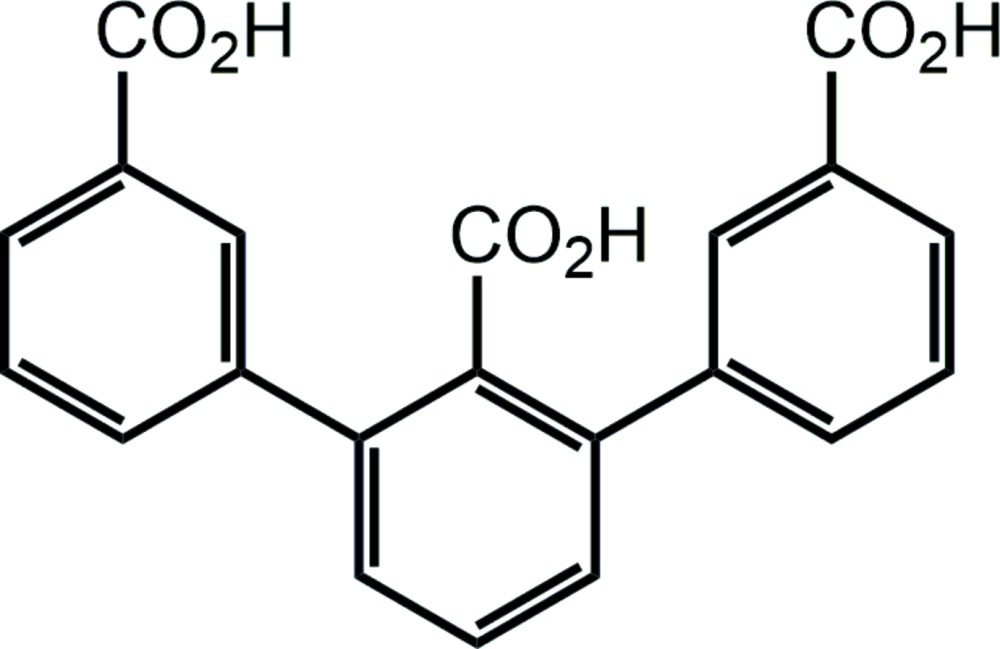


## Experimental   

### Crystal data  

C_21_H_14_O_6_
*M* *_r_* = 362.32
Orthorhombic, 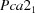

*a* = 23.1735 (9) Å
*b* = 7.2480 (2) Å
*c* = 20.3320 (8) Å
*V* = 3415.0 (2) Å^3^
*Z* = 8
Mo *K*α radiation
μ = 0.10 mm^−1^
*T* = 100 K
0.3 × 0.05 × 0.05 mm

### Data collection  

Bruker D8 VENTURE DUO diffractometer
Absorption correction: multi-scan (*SADABS*; Bruker, 2012 ▸) *T*_min_ = 0.695, *T*_max_ = 0.745
46489 measured reflections
6467 independent reflections
5899 reflections with *I* > 2σ(*I*)
*R*_int_ = 0.028

### Refinement  

*R*[*F* ^2^ > 2σ(*F* ^2^)] = 0.037
*wR*(*F* ^2^) = 0.091
*S* = 1.05
6467 reflections
511 parameters
2 restraints
H atoms treated by a mixture of independent and constrained refinement
Δρ_max_ = 0.27 e Å^−3^
Δρ_min_ = −0.17 e Å^−3^

### 

Data collection: *APEX2* (Bruker, 2012 ▸); cell refinement: *SAINT* (Bruker, 2012 ▸); data reduction: *SAINT*; program(s) used to solve structure: *SHELXT* (Sheldrick, 2015*a* ▸); program(s) used to refine structure: *SHELXL2014* (Sheldrick, 2015*b* ▸); molecular graphics: *OLEX2* (Dolomanov *et al.*, 2009 ▸); software used to prepare material for publication: *OLEX2*.

## Supplementary Material

Crystal structure: contains datablock(s) I. DOI: 10.1107/S2056989015015029/ld2133sup1.cif

Structure factors: contains datablock(s) I. DOI: 10.1107/S2056989015015029/ld2133Isup2.hkl

Supporting information file. DOI: 10.1107/S2056989015015029/ld2133Isup3.cml

**Fig. 1.**
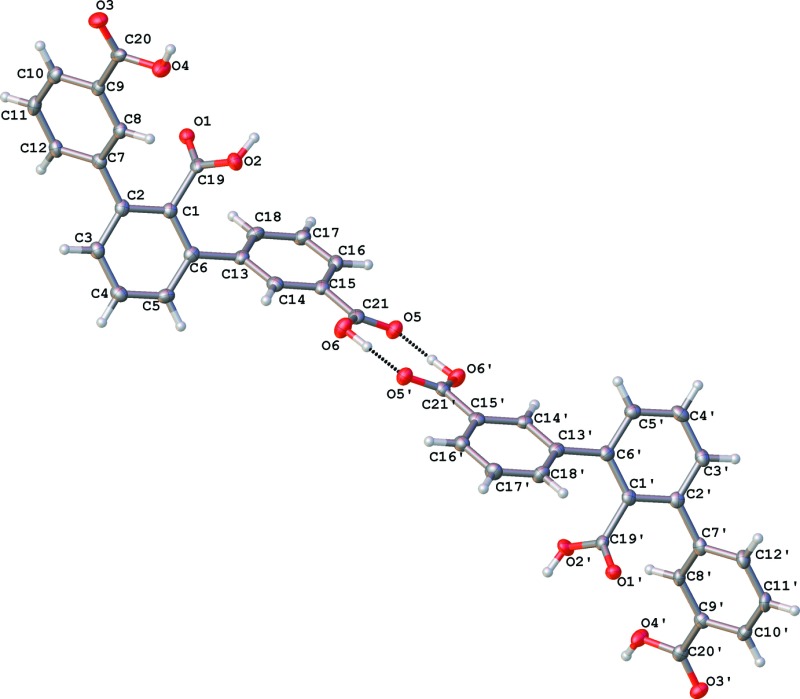
: The asymmetric unit of the title compound, with displacement elipsoids drawn at 50% probability level. Hydrogen atoms presented by spheres of an arbitrary radius. Intra-dimer hydrogen bonds are represented by dotted lines.

**Fig. 2.**
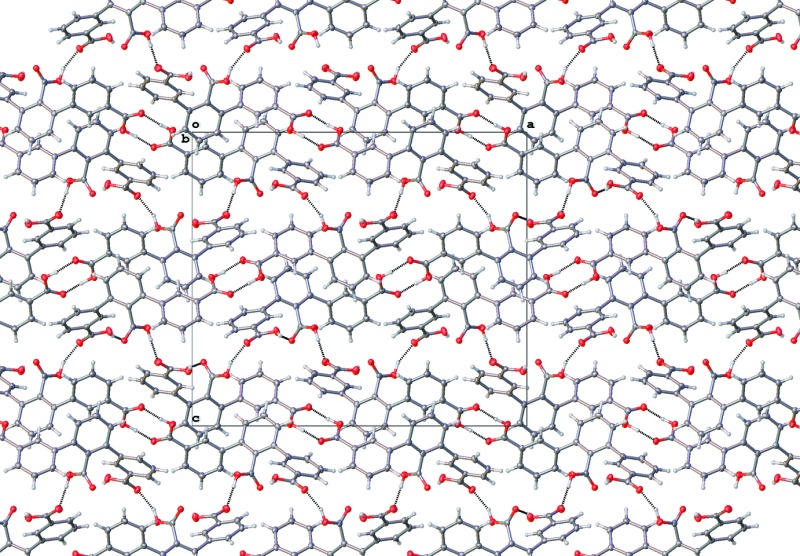
: Packing view along b axis. Hydrogen bonds are represented by dotted lines.

. DOI: 10.1107/S2056989015015029/ld2133fig1.tif
The asymmetric unit of the title compound, with displacement elipsoids drawn at 50% probability level. Hydrogen atoms presented by spheres of an arbitrary radius. Intra-dimer hydrogen bonds are represented by dotted lines.

b . DOI: 10.1107/S2056989015015029/ld2133fig2.tif
Packing view along *b* axis. Hydrogen bonds are represented by dotted lines.

CCDC reference: 1418223

Additional supporting information:  crystallographic information; 3D view; checkCIF report

## Figures and Tables

**Table 1:** Hydrogen-bond geometry (, )

*D*H*A*	*D*H	H*A*	*D* *A*	*D*H*A*
O2H2O3^i^	0.91(5)	1.79(5)	2.657(3)	159(4)
O4H4O1^ii^	0.90(6)	1.84(6)	2.689(3)	156(5)
O2H2O3^iii^	0.87(6)	1.76(6)	2.628(3)	171(5)
O4H4O1^iv^	0.84(5)	1.90(5)	2.717(3)	166(4)
O6H6O5	0.92(4)	1.69(4)	2.593(3)	168(4)
O6H6O5	1.01(3)	1.58(3)	2.581(3)	172(6)

Symmetry codes: (i) 
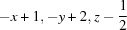
; (ii) 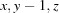; (iii) 
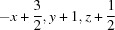
; (iv) 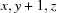.

## supplementary crystallographic information

### S1.1. Synthesis and crystallization

3,3''-di­methyl-[1,1':3',1''-terphenyl]-2'-carb­oxy­lic acid (0.432 g, 1.4 mmol) synthesized according to Du *et al.* (1986). The starting material was dissolved in 15 ml pyridine and brought to reflux. KMnO_4_ (0.50 g, 3.1 mmol) in 1 ml of water was added, and allowed to react for 2 hours. Subsequently, 3 more additions of KMnO_4_ (0.25 g, 1.6 mmol) in 1.5 ml of water were added every 2 hours for a total of 4 additions. After six hours, 10 ml of water was added and the reaction was refluxed overnight. The reaction mixture was filtered while hot to remove solid MnO_2_. The filtrate was then concentrated under reduced pressure, and treated by *12M* HCl. The resulting white precipitate was then filtered and purified via silica gel column chromatograph (50/50 hexanes/ethyl acetate with 0.5% acetic acid) to give of the title compound. (0.366 g *Yield 70%*) ^1^H NMR (DMSO, 400 MHz): δ 7.46 (d, 2H *J* = 8 Hz), 7.58 (t, 2H, *J* = 8 Hz), 7.61 (t, 1H, *J* = 7.2 Hz), 7.68 (d, 2H, *J* = 8Hz), 7.97 (d, 2H, *J* = 8 Hz), 8.01 (s, 2H), 13.01 (s, O-H). ^13^C NMR (DMSO, 100 MHz): δ 128.4, 128.7, 129.1, 129.2, 129.3, 130.8, 132.7, 133.9, 138.0, 140.39, 167.0, 169.7. Single crystals suitable for X-ray diffraction were obtained by vapor diffusion of hexane into an ethyl acetate solution of the title compound.

### S1.2. Refinement

All H atoms were located in difference Fourier maps but finally their positions were determined geometrically, except for the carb­oxy H atoms that were refined with isotropic thermal parameters. The O6—H6 bond length in the carb­oxy­lic acid dimer was restrained at the distance from the corresponding residual electron density peak to the oxygen (0.996 (2)Å) . This was done due to unreasonable lengthening (> 1.4 Å) of the H—O bond during the refinement. All other H atoms were refined using a riding model with fixed isotropic displacement parameters [Uiso(H) = 1.2Ueq(C) for the C(H) groups]. Additional crystal data, data collection and structure refinement details are summarized in Table 1.

### Crystal data

**Table:**

C_21_H_14_O_6_	*D*_x_ = 1.409 Mg m^−^^3^
*M**_r_* = 362.32	Mo *K*α radiation, λ = 0.71073 Å
Orthorhombic, *P**c**a*2_1_	Cell parameters from 9141 reflections
*a* = 23.1735 (9) Å	θ = 3.1–25.7°
*b* = 7.2480 (2) Å	µ = 0.10 mm^−^^1^
*c* = 20.3320 (8) Å	*T* = 100 K
*V* = 3415.0 (2) Å^3^	Needle, clear
*Z* = 8	0.3 × 0.05 × 0.05 mm
*F*(000) = 1504	

### Data collection

**Table:**

Bruker D8 VENTURE DUO diffractometer	6467 independent reflections
Radiation source: sealed tube, fine-focus	5899 reflections with *I* > 2σ(*I*)
TRIUMPH graphite monochromator	*R*_int_ = 0.028
Detector resolution: 10.5 pixels mm^-1^	θ_max_ = 25.7°, θ_min_ = 3.0°
ω and φ scans	*h* = −28→28
Absorption correction: multi-scan (*SADABS*; Bruker, 2012)	*k* = −7→8
*T*_min_ = 0.695, *T*_max_ = 0.745	*l* = −24→24
46489 measured reflections	

### Refinement

**Table:**

Refinement on *F*^2^	Primary atom site location: structure-invariant direct methods
Least-squares matrix: full	Hydrogen site location: mixed
*R*[*F*^2^ > 2σ(*F*^2^)] = 0.037	H atoms treated by a mixture of independent and constrained refinement
*wR*(*F*^2^) = 0.091	*w* = 1/[σ^2^(*F*_o_^2^) + (0.0518*P*)^2^ + 1.0855*P*] where *P* = (*F*_o_^2^ + 2*F*_c_^2^)/3
*S* = 1.05	(Δ/σ)_max_ < 0.001
6467 reflections	Δρ_max_ = 0.27 e Å^−^^3^
511 parameters	Δρ_min_ = −0.17 e Å^−^^3^
2 restraints	

### Special details

**Table:**

Experimental. SADABS-2012/1 (Bruker,2012) was used for absorption correction. wR2(int) was 0.0509 before and 0.0455 after correction. The Ratio of minimum to maximum transmission is 0.9326. The λ/2 correction factor is 0.0015.
Geometry. All e.s.d.'s (except the e.s.d. in the dihedral angle between two l.s. planes) are estimated using the full covariance matrix. The cell e.s.d.'s are taken into account individually in the estimation of e.s.d.'s in distances, angles and torsion angles; correlations between e.s.d.'s in cell parameters are only used when they are defined by crystal symmetry. An approximate (isotropic) treatment of cell e.s.d.'s is used for estimating e.s.d.'s involving l.s. planes.

### Fractional atomic coordinates and isotropic or equivalent isotropic displacement parameters (Å^2^)

**Table:**

	*x*	*y*	*z*	*U*_iso_*/*U*_eq_	
O1	0.96578 (9)	0.7615 (3)	0.28358 (11)	0.0228 (5)	
O2	0.89715 (9)	0.5814 (3)	0.32756 (11)	0.0244 (5)	
H2	0.8852 (17)	0.572 (5)	0.285 (2)	0.042 (11)*	
O3	1.10167 (9)	0.0388 (3)	0.28278 (11)	0.0303 (5)	
O4	1.01069 (10)	0.0942 (3)	0.31188 (12)	0.0306 (5)	
H4	1.006 (2)	−0.025 (8)	0.301 (3)	0.085 (18)*	
O5	0.65152 (9)	0.9096 (3)	0.43665 (11)	0.0258 (5)	
O6	0.70682 (9)	0.7133 (3)	0.49305 (12)	0.0285 (5)	
C1	0.95397 (12)	0.7560 (4)	0.40061 (15)	0.0188 (6)	
C2	1.00836 (13)	0.7148 (4)	0.42736 (15)	0.0200 (6)	
C3	1.02024 (14)	0.7721 (4)	0.49135 (16)	0.0214 (7)	
H3	1.0572	0.7495	0.5098	0.026*	
C4	0.97881 (14)	0.8613 (4)	0.52817 (16)	0.0235 (7)	
H4A	0.9872	0.8968	0.5721	0.028*	
C5	0.92531 (13)	0.8995 (4)	0.50187 (15)	0.0228 (7)	
H5	0.8969	0.9586	0.5282	0.027*	
C6	0.91246 (13)	0.8522 (4)	0.43706 (15)	0.0197 (6)	
C7	1.05206 (13)	0.6053 (4)	0.38977 (15)	0.0188 (6)	
C8	1.03776 (13)	0.4318 (4)	0.36459 (15)	0.0201 (7)	
H8	0.9999	0.3841	0.3704	0.024*	
C9	1.07890 (13)	0.3293 (4)	0.33113 (14)	0.0178 (6)	
C10	1.13447 (14)	0.3978 (4)	0.32208 (16)	0.0239 (7)	
H10	1.1625	0.3272	0.2991	0.029*	
C11	1.14841 (13)	0.5694 (4)	0.34687 (16)	0.0251 (7)	
H11	1.1861	0.6178	0.3404	0.030*	
C12	1.10790 (13)	0.6712 (4)	0.38102 (15)	0.0221 (6)	
H12	1.1183	0.7878	0.3988	0.026*	
C13	0.85570 (13)	0.9045 (4)	0.40774 (15)	0.0203 (6)	
C14	0.80514 (13)	0.8518 (4)	0.43914 (15)	0.0212 (7)	
H14	0.8072	0.7839	0.4790	0.025*	
C15	0.75168 (13)	0.8969 (4)	0.41302 (16)	0.0205 (6)	
C16	0.74787 (13)	0.9963 (4)	0.35433 (16)	0.0226 (7)	
H16	0.7113	1.0245	0.3357	0.027*	
C17	0.79797 (13)	1.0528 (4)	0.32379 (16)	0.0243 (7)	
H17	0.7959	1.1237	0.2846	0.029*	
C18	0.85140 (13)	1.0069 (4)	0.34989 (16)	0.0233 (7)	
H18	0.8855	1.0459	0.3280	0.028*	
C19	0.94049 (13)	0.7010 (4)	0.33133 (15)	0.0199 (6)	
C20	1.06555 (13)	0.1399 (4)	0.30611 (14)	0.0209 (6)	
C21	0.69880 (13)	0.8403 (4)	0.44841 (16)	0.0220 (6)	
O1'	0.29732 (9)	0.7389 (3)	0.70997 (10)	0.0213 (5)	
O2'	0.36530 (10)	0.9209 (3)	0.66648 (12)	0.0265 (5)	
H2'	0.376 (2)	0.949 (7)	0.706 (3)	0.070 (15)*	
O3'	0.16368 (9)	1.4521 (3)	0.71744 (11)	0.0247 (5)	
O4'	0.25240 (10)	1.4042 (3)	0.67837 (12)	0.0282 (5)	
H4'	0.2621 (18)	1.507 (7)	0.693 (2)	0.054 (13)*	
O5'	0.61435 (9)	0.6032 (3)	0.55199 (11)	0.0247 (5)	
O6'	0.55852 (10)	0.7980 (3)	0.49534 (11)	0.0271 (5)	
C1'	0.31076 (12)	0.7424 (4)	0.59342 (15)	0.0191 (6)	
C2'	0.25613 (13)	0.7813 (4)	0.56681 (15)	0.0193 (6)	
C3'	0.24393 (13)	0.7237 (4)	0.50299 (16)	0.0216 (7)	
H3'	0.2067	0.7449	0.4850	0.026*	
C4'	0.28570 (14)	0.6353 (4)	0.46537 (16)	0.0245 (7)	
H4'A	0.2771	0.5986	0.4216	0.029*	
C5'	0.33996 (13)	0.6003 (4)	0.49149 (16)	0.0214 (6)	
H5'	0.3685	0.5418	0.4652	0.026*	
C6'	0.35278 (13)	0.6504 (4)	0.55605 (15)	0.0198 (6)	
C7'	0.21240 (13)	0.8890 (4)	0.60456 (16)	0.0209 (7)	
C8'	0.22618 (12)	1.0626 (4)	0.63010 (14)	0.0183 (6)	
H8'	0.2640	1.1105	0.6247	0.022*	
C9'	0.18463 (13)	1.1658 (4)	0.66341 (15)	0.0198 (6)	
C10'	0.12932 (13)	1.0961 (4)	0.67205 (15)	0.0212 (6)	
H10'	0.1011	1.1663	0.6949	0.025*	
C11'	0.11546 (13)	0.9241 (4)	0.64717 (17)	0.0242 (7)	
H11'	0.0777	0.8760	0.6532	0.029*	
C12'	0.15643 (13)	0.8211 (4)	0.61342 (16)	0.0228 (7)	
H12'	0.1464	0.7036	0.5962	0.027*	
C13'	0.41042 (13)	0.6027 (4)	0.58405 (15)	0.0198 (6)	
C14'	0.46007 (13)	0.6550 (4)	0.55043 (15)	0.0192 (6)	
H14'	0.4569	0.7180	0.5096	0.023*	
C15'	0.51443 (13)	0.6154 (4)	0.57626 (15)	0.0199 (6)	
C16'	0.51981 (13)	0.5223 (4)	0.63543 (15)	0.0231 (7)	
H16'	0.5569	0.4950	0.6528	0.028*	
C17'	0.47048 (13)	0.4694 (4)	0.66912 (16)	0.0252 (7)	
H17'	0.4738	0.4067	0.7100	0.030*	
C18'	0.41620 (13)	0.5075 (4)	0.64352 (16)	0.0252 (7)	
H18'	0.3827	0.4685	0.6666	0.030*	
C19'	0.32344 (12)	0.7990 (4)	0.66247 (15)	0.0183 (6)	
C20'	0.19858 (13)	1.3529 (4)	0.68902 (15)	0.0210 (6)	
C21'	0.56704 (13)	0.6716 (4)	0.53989 (14)	0.0197 (6)	
H6'	0.5939 (17)	0.824 (6)	0.477 (2)	0.045 (12)*	
H6	0.6693 (16)	0.681 (8)	0.515 (3)	0.098 (19)*	

### Atomic displacement parameters (Å^2^)

**Table:**

	*U*^11^	*U*^22^	*U*^33^	*U*^12^	*U*^13^	*U*^23^
O1	0.0208 (11)	0.0242 (11)	0.0235 (11)	0.0006 (8)	−0.0009 (9)	−0.0026 (9)
O2	0.0228 (12)	0.0250 (11)	0.0255 (13)	−0.0031 (9)	−0.0048 (10)	−0.0020 (9)
O3	0.0304 (12)	0.0302 (12)	0.0304 (13)	0.0075 (10)	0.0024 (10)	−0.0070 (10)
O4	0.0275 (13)	0.0232 (12)	0.0411 (14)	−0.0028 (10)	0.0041 (10)	−0.0084 (10)
O5	0.0209 (11)	0.0256 (11)	0.0309 (12)	0.0030 (9)	0.0016 (9)	0.0029 (10)
O6	0.0221 (12)	0.0292 (12)	0.0341 (13)	0.0023 (10)	0.0037 (11)	0.0100 (11)
C1	0.0199 (15)	0.0150 (14)	0.0215 (15)	−0.0009 (11)	−0.0003 (12)	0.0006 (11)
C2	0.0192 (15)	0.0149 (13)	0.0259 (16)	−0.0022 (11)	−0.0009 (12)	0.0019 (12)
C3	0.0224 (16)	0.0195 (15)	0.0225 (17)	−0.0004 (12)	−0.0040 (14)	0.0022 (13)
C4	0.0287 (18)	0.0191 (15)	0.0228 (16)	0.0006 (13)	−0.0033 (13)	−0.0010 (12)
C5	0.0252 (17)	0.0213 (15)	0.0218 (16)	−0.0007 (12)	0.0047 (13)	−0.0016 (13)
C6	0.0207 (15)	0.0149 (14)	0.0236 (15)	−0.0017 (12)	0.0019 (13)	0.0017 (12)
C7	0.0217 (16)	0.0163 (14)	0.0183 (15)	0.0021 (12)	−0.0029 (12)	0.0022 (12)
C8	0.0181 (15)	0.0199 (15)	0.0222 (16)	0.0020 (12)	−0.0025 (12)	0.0011 (12)
C9	0.0166 (15)	0.0189 (14)	0.0178 (14)	0.0028 (11)	−0.0020 (12)	0.0042 (11)
C10	0.0211 (17)	0.0269 (16)	0.0235 (16)	0.0045 (13)	−0.0009 (13)	0.0041 (13)
C11	0.0166 (16)	0.0287 (17)	0.0299 (18)	−0.0010 (12)	−0.0020 (13)	0.0061 (14)
C12	0.0203 (16)	0.0222 (15)	0.0237 (16)	−0.0014 (12)	−0.0056 (13)	0.0025 (12)
C13	0.0201 (15)	0.0173 (14)	0.0234 (16)	0.0029 (12)	0.0012 (13)	−0.0033 (12)
C14	0.0255 (17)	0.0171 (14)	0.0211 (16)	0.0024 (12)	−0.0009 (13)	−0.0009 (12)
C15	0.0206 (15)	0.0158 (13)	0.0250 (16)	0.0026 (12)	−0.0001 (12)	−0.0050 (12)
C16	0.0206 (15)	0.0211 (15)	0.0262 (17)	0.0044 (13)	−0.0033 (13)	−0.0026 (13)
C17	0.0252 (17)	0.0252 (15)	0.0225 (17)	0.0048 (13)	0.0026 (13)	−0.0007 (13)
C18	0.0238 (16)	0.0227 (15)	0.0234 (17)	0.0021 (14)	0.0065 (12)	0.0007 (13)
C19	0.0173 (15)	0.0159 (14)	0.0264 (16)	0.0051 (12)	−0.0016 (13)	−0.0044 (12)
C20	0.0226 (17)	0.0248 (15)	0.0153 (14)	0.0081 (13)	−0.0009 (12)	0.0002 (12)
C21	0.0227 (17)	0.0170 (14)	0.0261 (16)	0.0037 (12)	−0.0006 (13)	−0.0049 (12)
O1'	0.0212 (11)	0.0221 (11)	0.0207 (11)	−0.0001 (8)	0.0011 (9)	−0.0013 (9)
O2'	0.0268 (12)	0.0283 (11)	0.0244 (12)	−0.0108 (9)	−0.0009 (10)	−0.0065 (10)
O3'	0.0237 (11)	0.0205 (11)	0.0301 (12)	0.0016 (9)	0.0070 (10)	−0.0003 (9)
O4'	0.0217 (11)	0.0201 (11)	0.0428 (15)	−0.0052 (9)	0.0083 (10)	−0.0080 (11)
O5'	0.0205 (12)	0.0258 (11)	0.0277 (12)	0.0002 (9)	0.0022 (9)	0.0023 (10)
O6'	0.0232 (12)	0.0300 (12)	0.0280 (12)	0.0029 (9)	0.0047 (10)	0.0076 (10)
C1'	0.0191 (15)	0.0141 (13)	0.0241 (16)	−0.0039 (11)	−0.0004 (13)	0.0006 (12)
C2'	0.0209 (16)	0.0136 (13)	0.0233 (16)	−0.0028 (11)	−0.0002 (12)	0.0020 (12)
C3'	0.0221 (16)	0.0162 (14)	0.0264 (17)	−0.0011 (12)	−0.0064 (13)	0.0002 (12)
C4'	0.0330 (18)	0.0204 (16)	0.0201 (15)	−0.0046 (13)	−0.0039 (13)	−0.0020 (12)
C5'	0.0235 (17)	0.0177 (14)	0.0229 (15)	−0.0012 (12)	0.0020 (13)	−0.0020 (12)
C6'	0.0204 (16)	0.0157 (13)	0.0234 (15)	−0.0037 (11)	0.0000 (12)	−0.0017 (12)
C7'	0.0201 (16)	0.0197 (14)	0.0227 (16)	−0.0001 (12)	−0.0060 (13)	0.0049 (12)
C8'	0.0140 (15)	0.0198 (15)	0.0212 (16)	−0.0025 (11)	−0.0027 (12)	0.0031 (12)
C9'	0.0228 (15)	0.0182 (14)	0.0185 (14)	−0.0001 (12)	−0.0024 (13)	0.0029 (12)
C10'	0.0171 (15)	0.0237 (15)	0.0227 (15)	0.0025 (12)	0.0018 (13)	0.0025 (12)
C11'	0.0151 (16)	0.0265 (16)	0.0309 (18)	−0.0040 (12)	−0.0045 (13)	0.0029 (14)
C12'	0.0203 (16)	0.0181 (14)	0.0301 (17)	−0.0029 (12)	−0.0057 (13)	−0.0005 (12)
C13'	0.0217 (15)	0.0152 (13)	0.0226 (16)	−0.0012 (12)	0.0011 (12)	−0.0024 (12)
C14'	0.0240 (16)	0.0155 (13)	0.0180 (14)	0.0003 (11)	0.0029 (12)	−0.0023 (12)
C15'	0.0245 (16)	0.0146 (14)	0.0205 (15)	0.0011 (12)	0.0003 (12)	−0.0041 (12)
C16'	0.0241 (16)	0.0230 (15)	0.0221 (16)	0.0028 (13)	−0.0014 (12)	−0.0017 (12)
C17'	0.0275 (17)	0.0275 (16)	0.0207 (16)	0.0011 (13)	−0.0002 (13)	0.0040 (14)
C18'	0.0226 (16)	0.0256 (16)	0.0272 (18)	0.0000 (14)	0.0057 (13)	0.0026 (14)
C19'	0.0151 (14)	0.0174 (13)	0.0223 (15)	0.0017 (11)	−0.0002 (13)	−0.0004 (12)
C20'	0.0211 (16)	0.0202 (14)	0.0217 (15)	−0.0021 (13)	0.0026 (13)	0.0052 (12)
C21'	0.0224 (16)	0.0167 (13)	0.0198 (15)	−0.0017 (12)	−0.0019 (12)	−0.0023 (12)

### Geometric parameters (Å, º)

**Table:**

O1—C19	1.216 (4)	O1'—C19'	1.220 (4)
O2—H2	0.91 (5)	O2'—H2'	0.87 (6)
O2—C19	1.329 (4)	O2'—C19'	1.314 (4)
O3—C20	1.209 (4)	O3'—C20'	1.227 (4)
O4—H4	0.90 (6)	O4'—H4'	0.84 (5)
O4—C20	1.319 (4)	O4'—C20'	1.319 (4)
O5—C21	1.229 (4)	O5'—C21'	1.228 (4)
O6—C21	1.306 (4)	O6'—C21'	1.303 (4)
O6—H6	1.01 (3)	O6'—H6'	0.92 (4)
C1—C2	1.405 (4)	C1'—C2'	1.405 (4)
C1—C6	1.400 (4)	C1'—C6'	1.404 (4)
C1—C19	1.497 (4)	C1'—C19'	1.492 (4)
C2—C3	1.393 (5)	C2'—C3'	1.392 (4)
C2—C7	1.496 (4)	C2'—C7'	1.491 (4)
C3—H3	0.9500	C3'—H3'	0.9500
C3—C4	1.378 (4)	C3'—C4'	1.390 (4)
C4—H4A	0.9500	C4'—H4'A	0.9500
C4—C5	1.378 (4)	C4'—C5'	1.388 (4)
C5—H5	0.9500	C5'—H5'	0.9500
C5—C6	1.394 (5)	C5'—C6'	1.394 (4)
C6—C13	1.493 (4)	C6'—C13'	1.493 (4)
C7—C8	1.398 (4)	C7'—C8'	1.398 (4)
C7—C12	1.391 (4)	C7'—C12'	1.399 (4)
C8—H8	0.9500	C8'—H8'	0.9500
C8—C9	1.387 (4)	C8'—C9'	1.395 (4)
C9—C10	1.393 (4)	C9'—C10'	1.389 (4)
C9—C20	1.496 (4)	C9'—C20'	1.488 (4)
C10—H10	0.9500	C10'—H10'	0.9500
C10—C11	1.380 (5)	C10'—C11'	1.384 (4)
C11—H11	0.9500	C11'—H11'	0.9500
C11—C12	1.381 (5)	C11'—C12'	1.389 (5)
C12—H12	0.9500	C12'—H12'	0.9500
C13—C14	1.388 (4)	C13'—C14'	1.391 (4)
C13—C18	1.394 (5)	C13'—C18'	1.399 (4)
C14—H14	0.9500	C14'—H14'	0.9500
C14—C15	1.387 (4)	C14'—C15'	1.394 (4)
C15—C16	1.397 (5)	C15'—C16'	1.385 (4)
C15—C21	1.479 (5)	C15'—C21'	1.483 (4)
C16—H16	0.9500	C16'—H16'	0.9500
C16—C17	1.379 (5)	C16'—C17'	1.387 (4)
C17—H17	0.9500	C17'—H17'	0.9500
C17—C18	1.387 (4)	C17'—C18'	1.389 (4)
C18—H18	0.9500	C18'—H18'	0.9500
			
C19—O2—H2	110 (3)	C19'—O2'—H2'	115 (3)
C20—O4—H4	109 (3)	C20'—O4'—H4'	117 (3)
C21—O6—H6	111 (3)	C21'—O6'—H6'	107 (3)
C2—C1—C19	119.7 (3)	C2'—C1'—C19'	119.0 (3)
C6—C1—C2	121.1 (3)	C6'—C1'—C2'	120.8 (3)
C6—C1—C19	119.2 (3)	C6'—C1'—C19'	120.2 (3)
C1—C2—C7	121.5 (3)	C1'—C2'—C7'	121.3 (3)
C3—C2—C1	118.4 (3)	C3'—C2'—C1'	118.8 (3)
C3—C2—C7	120.1 (3)	C3'—C2'—C7'	119.9 (3)
C2—C3—H3	119.7	C2'—C3'—H3'	119.7
C4—C3—C2	120.6 (3)	C4'—C3'—C2'	120.7 (3)
C4—C3—H3	119.7	C4'—C3'—H3'	119.7
C3—C4—H4A	119.7	C3'—C4'—H4'A	119.9
C5—C4—C3	120.7 (3)	C5'—C4'—C3'	120.3 (3)
C5—C4—H4A	119.7	C5'—C4'—H4'A	119.9
C4—C5—H5	119.7	C4'—C5'—H5'	119.8
C4—C5—C6	120.6 (3)	C4'—C5'—C6'	120.4 (3)
C6—C5—H5	119.7	C6'—C5'—H5'	119.8
C1—C6—C13	121.3 (3)	C1'—C6'—C13'	121.6 (3)
C5—C6—C1	118.4 (3)	C5'—C6'—C1'	119.1 (3)
C5—C6—C13	120.2 (3)	C5'—C6'—C13'	119.3 (3)
C8—C7—C2	120.2 (3)	C8'—C7'—C2'	120.5 (3)
C12—C7—C2	120.9 (3)	C8'—C7'—C12'	118.7 (3)
C12—C7—C8	118.9 (3)	C12'—C7'—C2'	120.8 (3)
C7—C8—H8	120.0	C7'—C8'—H8'	119.8
C9—C8—C7	119.9 (3)	C9'—C8'—C7'	120.4 (3)
C9—C8—H8	120.0	C9'—C8'—H8'	119.8
C8—C9—C10	120.6 (3)	C8'—C9'—C20'	120.6 (3)
C8—C9—C20	121.1 (3)	C10'—C9'—C8'	120.2 (3)
C10—C9—C20	118.3 (3)	C10'—C9'—C20'	119.2 (3)
C9—C10—H10	120.3	C9'—C10'—H10'	120.1
C11—C10—C9	119.3 (3)	C11'—C10'—C9'	119.7 (3)
C11—C10—H10	120.3	C11'—C10'—H10'	120.1
C10—C11—H11	119.8	C10'—C11'—H11'	119.8
C10—C11—C12	120.4 (3)	C10'—C11'—C12'	120.4 (3)
C12—C11—H11	119.8	C12'—C11'—H11'	119.8
C7—C12—H12	119.5	C7'—C12'—H12'	119.7
C11—C12—C7	120.9 (3)	C11'—C12'—C7'	120.6 (3)
C11—C12—H12	119.5	C11'—C12'—H12'	119.7
C14—C13—C6	119.3 (3)	C14'—C13'—C6'	119.3 (3)
C14—C13—C18	118.3 (3)	C14'—C13'—C18'	118.7 (3)
C18—C13—C6	122.3 (3)	C18'—C13'—C6'	122.0 (3)
C13—C14—H14	119.6	C13'—C14'—H14'	119.8
C15—C14—C13	120.9 (3)	C13'—C14'—C15'	120.4 (3)
C15—C14—H14	119.6	C15'—C14'—H14'	119.8
C14—C15—C16	120.3 (3)	C14'—C15'—C21'	119.9 (3)
C14—C15—C21	119.2 (3)	C16'—C15'—C14'	120.6 (3)
C16—C15—C21	120.4 (3)	C16'—C15'—C21'	119.5 (3)
C15—C16—H16	120.5	C15'—C16'—H16'	120.3
C17—C16—C15	119.0 (3)	C15'—C16'—C17'	119.3 (3)
C17—C16—H16	120.5	C17'—C16'—H16'	120.3
C16—C17—H17	119.7	C16'—C17'—H17'	119.8
C16—C17—C18	120.5 (3)	C16'—C17'—C18'	120.4 (3)
C18—C17—H17	119.7	C18'—C17'—H17'	119.8
C13—C18—H18	119.5	C13'—C18'—H18'	119.7
C17—C18—C13	120.9 (3)	C17'—C18'—C13'	120.6 (3)
C17—C18—H18	119.5	C17'—C18'—H18'	119.7
O1—C19—O2	123.6 (3)	O1'—C19'—O2'	123.8 (3)
O1—C19—C1	123.7 (3)	O1'—C19'—C1'	123.3 (3)
O2—C19—C1	112.7 (3)	O2'—C19'—C1'	112.9 (3)
O3—C20—O4	123.3 (3)	O3'—C20'—O4'	122.4 (3)
O3—C20—C9	123.1 (3)	O3'—C20'—C9'	123.8 (3)
O4—C20—C9	113.6 (2)	O4'—C20'—C9'	113.9 (3)
O5—C21—O6	123.4 (3)	O5'—C21'—O6'	123.9 (3)
O5—C21—C15	122.1 (3)	O5'—C21'—C15'	121.5 (3)
O6—C21—C15	114.6 (3)	O6'—C21'—C15'	114.5 (3)
			
C1—C2—C3—C4	2.2 (4)	C1'—C2'—C3'—C4'	−2.3 (4)
C1—C2—C7—C8	−54.7 (4)	C1'—C2'—C7'—C8'	54.2 (4)
C1—C2—C7—C12	127.4 (3)	C1'—C2'—C7'—C12'	−127.7 (3)
C1—C6—C13—C14	125.4 (3)	C1'—C6'—C13'—C14'	−127.7 (3)
C1—C6—C13—C18	−55.3 (4)	C1'—C6'—C13'—C18'	52.0 (4)
C2—C1—C6—C5	−2.8 (4)	C2'—C1'—C6'—C5'	1.0 (4)
C2—C1—C6—C13	176.9 (3)	C2'—C1'—C6'—C13'	−178.1 (3)
C2—C1—C19—O1	−63.6 (4)	C2'—C1'—C19'—O1'	62.6 (4)
C2—C1—C19—O2	117.5 (3)	C2'—C1'—C19'—O2'	−117.0 (3)
C2—C3—C4—C5	−1.6 (5)	C2'—C3'—C4'—C5'	1.2 (4)
C2—C7—C8—C9	−178.5 (3)	C2'—C7'—C8'—C9'	177.8 (3)
C2—C7—C12—C11	179.4 (3)	C2'—C7'—C12'—C11'	−178.3 (3)
C3—C2—C7—C8	122.8 (3)	C3'—C2'—C7'—C8'	−123.3 (3)
C3—C2—C7—C12	−55.2 (4)	C3'—C2'—C7'—C12'	54.8 (4)
C3—C4—C5—C6	−1.4 (5)	C3'—C4'—C5'—C6'	1.1 (4)
C4—C5—C6—C1	3.5 (4)	C4'—C5'—C6'—C1'	−2.2 (4)
C4—C5—C6—C13	−176.2 (3)	C4'—C5'—C6'—C13'	176.9 (3)
C5—C6—C13—C14	−54.9 (4)	C5'—C6'—C13'—C14'	53.2 (4)
C5—C6—C13—C18	124.4 (3)	C5'—C6'—C13'—C18'	−127.1 (3)
C6—C1—C2—C3	0.0 (4)	C6'—C1'—C2'—C3'	1.2 (4)
C6—C1—C2—C7	177.4 (3)	C6'—C1'—C2'—C7'	−176.3 (3)
C6—C1—C19—O1	114.7 (3)	C6'—C1'—C19'—O1'	−116.9 (3)
C6—C1—C19—O2	−64.1 (3)	C6'—C1'—C19'—O2'	63.6 (4)
C6—C13—C14—C15	−179.5 (3)	C6'—C13'—C14'—C15'	178.7 (3)
C6—C13—C18—C17	179.8 (3)	C6'—C13'—C18'—C17'	−178.3 (3)
C7—C2—C3—C4	−175.3 (3)	C7'—C2'—C3'—C4'	175.2 (3)
C7—C8—C9—C10	−0.2 (4)	C7'—C8'—C9'—C10'	0.6 (4)
C7—C8—C9—C20	177.4 (3)	C7'—C8'—C9'—C20'	−178.2 (3)
C8—C7—C12—C11	1.4 (4)	C8'—C7'—C12'—C11'	−0.2 (5)
C8—C9—C10—C11	0.1 (4)	C8'—C9'—C10'—C11'	−0.3 (4)
C8—C9—C20—O3	−172.2 (3)	C8'—C9'—C20'—O3'	178.4 (3)
C8—C9—C20—O4	7.9 (4)	C8'—C9'—C20'—O4'	−1.5 (4)
C9—C10—C11—C12	0.8 (5)	C9'—C10'—C11'—C12'	−0.3 (5)
C10—C9—C20—O3	5.5 (4)	C10'—C9'—C20'—O3'	−0.5 (5)
C10—C9—C20—O4	−174.4 (3)	C10'—C9'—C20'—O4'	179.6 (3)
C10—C11—C12—C7	−1.5 (5)	C10'—C11'—C12'—C7'	0.5 (5)
C12—C7—C8—C9	−0.5 (4)	C12'—C7'—C8'—C9'	−0.3 (4)
C13—C14—C15—C16	0.1 (4)	C13'—C14'—C15'—C16'	0.5 (4)
C13—C14—C15—C21	−179.2 (3)	C13'—C14'—C15'—C21'	179.9 (3)
C14—C13—C18—C17	−0.9 (4)	C14'—C13'—C18'—C17'	1.4 (4)
C14—C15—C16—C17	−1.7 (4)	C14'—C15'—C16'—C17'	−0.4 (4)
C14—C15—C21—O5	163.2 (3)	C14'—C15'—C21'—O5'	−162.4 (3)
C14—C15—C21—O6	−16.1 (4)	C14'—C15'—C21'—O6'	18.1 (4)
C15—C16—C17—C18	1.9 (5)	C15'—C16'—C17'—C18'	0.7 (5)
C16—C15—C21—O5	−16.1 (4)	C16'—C15'—C21'—O5'	17.0 (4)
C16—C15—C21—O6	164.5 (3)	C16'—C15'—C21'—O6'	−162.6 (3)
C16—C17—C18—C13	−0.6 (5)	C16'—C17'—C18'—C13'	−1.2 (5)
C18—C13—C14—C15	1.1 (4)	C18'—C13'—C14'—C15'	−1.0 (4)
C19—C1—C2—C3	178.3 (3)	C19'—C1'—C2'—C3'	−178.2 (3)
C19—C1—C2—C7	−4.2 (4)	C19'—C1'—C2'—C7'	4.3 (4)
C19—C1—C6—C5	178.9 (3)	C19'—C1'—C6'—C5'	−179.5 (3)
C19—C1—C6—C13	−1.4 (4)	C19'—C1'—C6'—C13'	1.3 (4)
C20—C9—C10—C11	−177.6 (3)	C20'—C9'—C10'—C11'	178.6 (3)
C21—C15—C16—C17	177.7 (3)	C21'—C15'—C16'—C17'	−179.7 (3)

### Hydrogen-bond geometry (Å, º)

**Table:**

*D*—H···*A*	*D*—H	H···*A*	*D*···*A*	*D*—H···*A*
O2—H2···O3′^i^	0.91 (5)	1.79 (5)	2.657 (3)	159 (4)
O4—H4···O1^ii^	0.90 (6)	1.84 (6)	2.689 (3)	156 (5)
O2′—H2′···O3^iii^	0.87 (6)	1.76 (6)	2.628 (3)	171 (5)
O4′—H4′···O1′^iv^	0.84 (5)	1.90 (5)	2.717 (3)	166 (4)
O6′—H6′···O5	0.92 (4)	1.69 (4)	2.593 (3)	168 (4)
O6—H6···O5′	1.01 (3)	1.58 (3)	2.581 (3)	172 (6)

Symmetry codes: (i) −*x*+1, −*y*+2, *z*−1/2; (ii) *x*, *y*−1, *z*; (iii) −*x*+3/2, *y*+1, *z*+1/2; (iv) *x*, *y*+1, *z*.
