# Supplementary figures and images for: Crystal structure of [1,1′:3′,1′′-ter­phenyl]-2′,3,3′′-tri­carb­oxy­lic acid
Source: Acta Crystallogr E Crystallogr Commun. 2015 Aug 22;71(Pt 9):o667–8. doi: 10.1107/S2056989015015029 (PMC4555404; doi:10.1107/S2056989015015029)

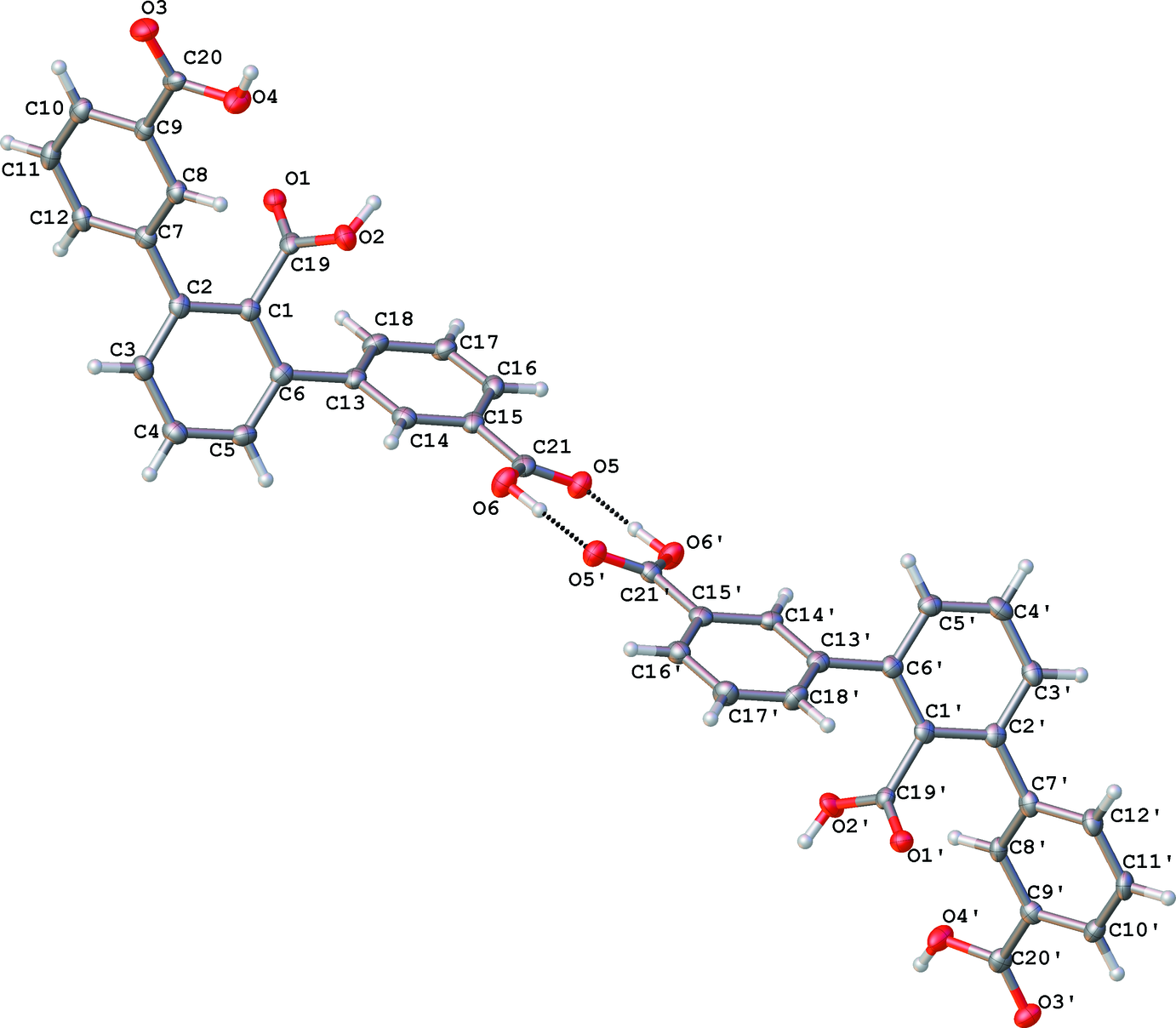

Supplement: Supplementary file 4 [file e-71-0o667-fig1.tif]

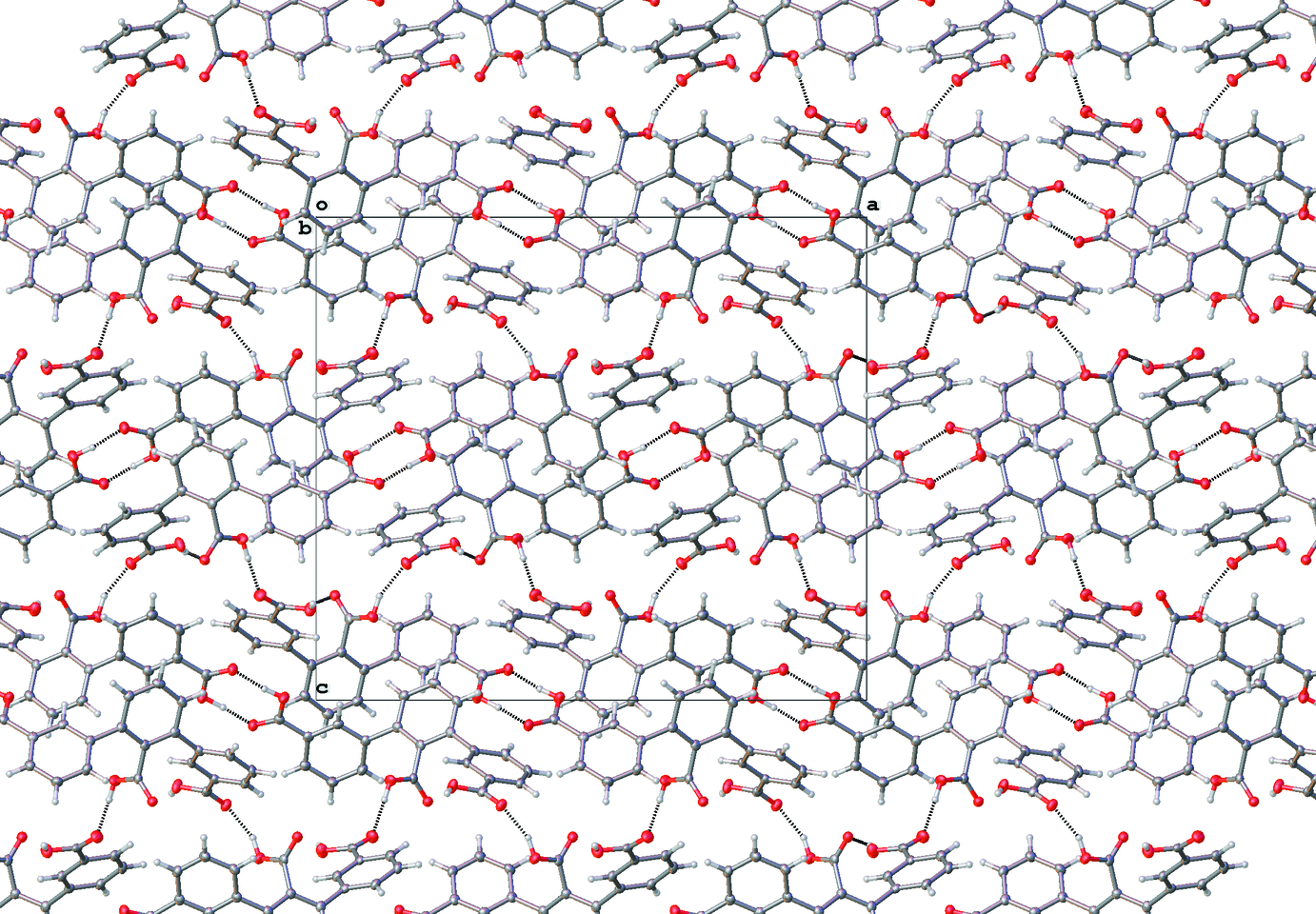

Supplement: Supplementary file 5 [file e-71-0o667-fig2.tif]
